# Beyond 30-day mortality: causes and risk factors of half-year postdischarge mortality after gastric cancer surgery

**DOI:** 10.1007/s10120-026-01750-x

**Published:** 2026-10-21

**Authors:** Kyoyoung Park, Hyuk-Joon Lee, Han-Kwang Yang, Min Kyu Kang, Jane Chungyoon Kim, Sa-Hong Kim, Jeesun Kim, Seong-Ho Kong, Yun-Suhk Suh, Do Joong Park

**Affiliations:** 1 Department of Surgery, Seoul National University Hospital, Seoul, Korea; 2 Department of Surgery, Seoul National University College of Medicine, Seoul, Korea; 3 Department of Surgery and Cancer Research Institute, Seoul National University College of Medicine, 101, Daehak-ro, Jongno-gu, Seoul, 110-744 Korea; 4 Department of Surgery, Seoul National University Bundang Hospital, Seongnam, Korea; 5 Present Address: Division of Gastrointestinal Surgery, Ewha Womans University Seoul Hospital, Seoul, Republic of Korea; 6 Present Address: National Cancer Center, Goyang, Republic of Korea

**Keywords:** Gastric neoplasms, Gastrectomy, Post-discharge mortality, Risk factors

## Abstract

**Background:**

Conventional 30-day or in-hospital mortality underestimates the true burden of postoperative death after gastrectomy. This study aimed to characterize post-discharge mortality and its evolving determinants following gastric cancer surgery.

**Methods:**

We retrospectively analyzed patients who underwent gastrectomy for primary gastric cancer at Seoul National University Hospital between 2013 and 2019. Mortality data were updated through national death registry linkage with complete follow-up of at least 5 years. In-hospital deaths were reviewed using medical records, while out-of-hospital deaths were classified using death certificates and family interviews. Cox regression analyses were performed across sequential postoperative intervals up to 5 years.

**Results:**

Among 4802 patients included in the analysis, overall mortality within 6 months was 1.2% (*n* = 57), of which 89.5% occurred after discharge. Thirty-day and 90-day mortality were 0.1% and 0.5%, respectively, demonstrating substantial underestimation by conventional short-term endpoints. Early mortality was primarily associated with emergency surgery and postoperative complications. Mid-term mortality was strongly linked to psychiatric disorders and systemic comorbidities, while low body mass index emerged as a significant predictor from 6 months onward. With longer follow-up, older age, renal or hepatic disease, multiple comorbidities, and incomplete (non-R0) resection became dominant risk factors. Tumor stage showed a strong association with mortality at 3 years.

**Conclusions:**

Postoperative mortality after gastrectomy is a dynamic process extending far beyond hospital discharge and shaped by distinct risk factors over time. Because most early deaths occur after discharge, improved survival requires surveillance strategies that target high-risk patients beyond the conventional 30-day window.

**Supplementary Information:**

The online version contains supplementary material available at https://doi.org/10.1007/s10120-026-01750-x.

## Introduction

Gastric cancer remains a major global health burden, particularly in East Asia, where its incidence is among the highest worldwide [1]. In South Korea, it ranked fifth in both cancer incidence and mortality in 2022, accounting for 10.8% of all new cancers and 9.5% of cancer-related deaths [2]. Despite remarkable advances in surgical and perioperative management, postoperative mortality remains an important benchmark for surgical quality [3].

Gastrectomy is the standard treatment for gastric cancer [4]. However, as surgical candidates become older and more medically complex, accurate risk prediction for postoperative complications and mortality has gained increasing importance [5–9]. The concept of surgical volume proposed by Howard in 1979 established that higher-volume hospitals achieve lower mortality rates [10]. Consistent with this, outcomes vary widely across countries. National 30-day mortality rates after gastrectomy show substantial international variation. Several East Asian countries consistently report mortality rates below 1%, including China (0.3%) [11], Japan (0.5–1.2%) [12, 13], and South Korea, where high-volume centers report rates as low as 0.1% and nationwide registry data indicate an overall mortality of approximately 1% [14]. In contrast, higher 30-day mortality rates have been reported in Western countries, including 4.1% in the United States [15], 5.7% in Italy (with 90-day mortality reaching 10.7%) [16], 1.3–3.9% at high-volume centers in the United Kingdom [17], and approximately 8% in Germany [18].

Postoperative deaths continue to occur despite low reported short-term mortality rates. Most studies, however, have limited their assessment to 30-day or in-hospital mortality, thereby overlooking deaths occurring after discharge. Prior reports have shown that 90-day mortality can be 40% to twofold higher than 30-day mortality [19, 20], highlighting the inadequacy of short-term endpoints.

Therefore, it is essential to move beyond conventional metrics and examine postoperative mortality over extended intervals. By identifying time-specific causes and risk factors, clinicians may better understand delayed deaths and develop tailored strategies to prevent them. The present study aimed to analyze postoperative mortality beyond 30 days, elucidate its causes, and identify temporal predictors of death based on over seven years of data.

## Methods

### Patients and data

This retrospective study analyzed a prospectively maintained gastric cancer surgery database at Seoul National University Hospital. All patients who underwent gastrectomy for primary gastric cancer between January 2013 and September 2019 were included, regardless of surgical approach or R0 resection status. Both elective and emergency operations were analyzed, although emergency cases were rare. Patients who underwent exploration only, palliative bypass without resection, or remnant total gastrectomy for recurrence were excluded.

Postoperative mortality data were obtained and updated as of September 10, 2024, using records from the National Statistical Office, enabling complete 5-year follow-up for all patients. For in-hospital deaths, causes were identified through chart review. For out-of-hospital deaths, causes were primarily determined from National Statistical Office death certificate codes. Telephone interviews with family members were attempted when the cause was unclear, but since they seldom provided conclusive information, such cases were classified as “unknown.”

### Endpoints

The primary endpoint was to determine postoperative and post-discharge mortality rates at 30-day intervals up to 6 months and to investigate causes of post-discharge deaths often missed by conventional 30-day analyses.

The secondary endpoint was to evaluate the influence of comorbidities and postoperative complications on mortality and identify predictors of long-term postoperative death.

### Definitions

The pathological tumor stage was classified according to the 8th edition of the American Joint Committee on Cancer (AJCC) TNM staging system [21].

Comorbid conditions were identified from medical records at the time of surgery and categorized into predefined clinical domains, including cardiovascular, neurologic, psychiatric, pulmonary, hepatic, renal, and other systemic diseases. Each comorbidity was recorded as present or absent based on documented diagnoses or ongoing treatment. The overall burden of comorbidity was quantified using the Charlson Comorbidity Index (CCI) [22].

Conversion surgery was defined as gastrectomy performed after systemic chemotherapy in patients initially considered unresectable or metastatic, in whom potentially curative (R0) resection became feasible following multidisciplinary team evaluation. During the study period,* neoadjuvant chemotherapy for initially resectable gastric cancer was rare in our institution*,* and such cases were not classified as conversion surgery.*

Postoperative complications were assessed and graded using the Clavien–Dindo classification system [23]. Mortality data were collected using a multimodal approach: in-hospital deaths were identified through comprehensive medical chart review, while out-of-hospital deaths were ascertained through linkage with death certification records from the National Statistical Office. For cases in which the cause of death was unclear or discordant between sources, telephone interviews with family members were conducted to confirm the circumstances and primary cause of death.

Causes of death were classified into predefined categories based on information from medical records, death certification data, and family interviews. Deaths due to cancer progression were defined as those attributable to recurrence or progression of gastric cancer. Acute abdominal events included fatal intra-abdominal conditions such as bowel perforation, peritonitis, or postoperative anastomotic complications. Pneumonia referred to deaths primarily caused by respiratory infection. Neurologic causes included fatal cerebrovascular events or intracranial hemorrhage. Trauma-related deaths encompassed deaths due to external injury, while other malignancy referred to deaths attributable to non-gastric primary cancers. Cases in which a definitive cause of death could not be determined despite available information were classified as unknown.

### Discharge criteria

During the study period, patients were managed according to a standardized institutional clinical pathway, and discharge decisions were based on overall clinical stability, tolerance of oral intake, absence of unresolved complications, laboratory findings, and the need for continued inpatient care. In general, patients were allowed sips of water on postoperative day (POD) 3 and a semi-fluid diet on POD 4, and were discharged after suture removal on POD 7–8 if no clinical concerns were identified.

Comorbid conditions and functional status were considered when determining discharge timing. For example, in patients receiving anticoagulant or antiplatelet therapy, discharge was planned after safe resumption of medication without bleeding complications. Patients with psychiatric disorders were evaluated perioperatively in consultation with psychiatry, and for patients undergoing dialysis, discharge was coordinated after stabilization of the dialysis schedule. Discharge could also be delayed for elderly or frail patients or when transfer to another facility required coordination.

 Objective indicators previously reported to predict an uncomplicated postoperative course were also considered [24]. Patients younger than 68 years who underwent laparoscopic gastrectomy without combined resection, with POD4 body temperature ≤ 37.4 °C, POD5 C-reactive protein ≤ 5.38 mg/mL, and a POD5-to-preoperative white blood cell count ratio ≤ 1.2, were regarded as more likely to have a stable recovery and were assessed accordingly together with their clinical condition.

### Postoperative follow-up protocol

Patients were followed according to a standardized institutional surveillance protocol after discharge. The first outpatient visit was routinely scheduled approximately 2 weeks after surgery, followed by another visit at about 2 months. Thereafter, follow-up intervals were determined based on tumor stage and clinical condition. Patients with advanced gastric cancer were generally followed every 4 months during the first 3 postoperative years, whereas those with early gastric cancer were followed every 6 months during the same period. After 3 years, annual follow-up was typically performed.

At outpatient visits, routine evaluations included chest X-ray, erect abdominal X-ray, and laboratory tests such as complete blood count, inflammatory markers, liver and renal function tests, and tumor markers, with additional assessments including vitamin B12 or iron studies performed when clinically indicated. For surveillance imaging, computed tomography and abdominal ultrasonography were performed alternately at approximately 6-month intervals, and endoscopic surveillance of the remnant stomach was performed annually starting 1 year after surgery. Follow-up intervals and additional evaluations were adjusted at the discretion of the treating physician according to the patient’s clinical condition.

For patients with significant comorbidities who were followed by other specialties or external institutions, coordination was managed according to institutional practice. For patients followed within the same hospital, direct communication with the relevant specialty was conducted through formal interdepartmental consultation. For those followed at external institutions, patients were instructed to continue follow-up with their respective providers after discharge, and referral letters were provided when detailed communication regarding surgical findings or postoperative care was considered necessary.

### Statistical analysis

All analyses were performed using SPSS Statistics 29.0 (IBM Corp., Armonk, NY, USA). Categorical variables were compared using the χ² or Fisher’s exact test, and continuous variables using the t-test or Mann–Whitney U test. Univariate and multivariate Cox proportional hazards models identified time-specific mortality predictors, with hazard ratios (HRs) and 95% confidence intervals (CIs). Logistic regression was used for binary outcomes, and *p* < 0.05 was considered significant. Adjusted HRs were summarized as log-scale forest plots generated in R 4.5.1 (ggplot2 v3.5.2).

**Fig. 1:**
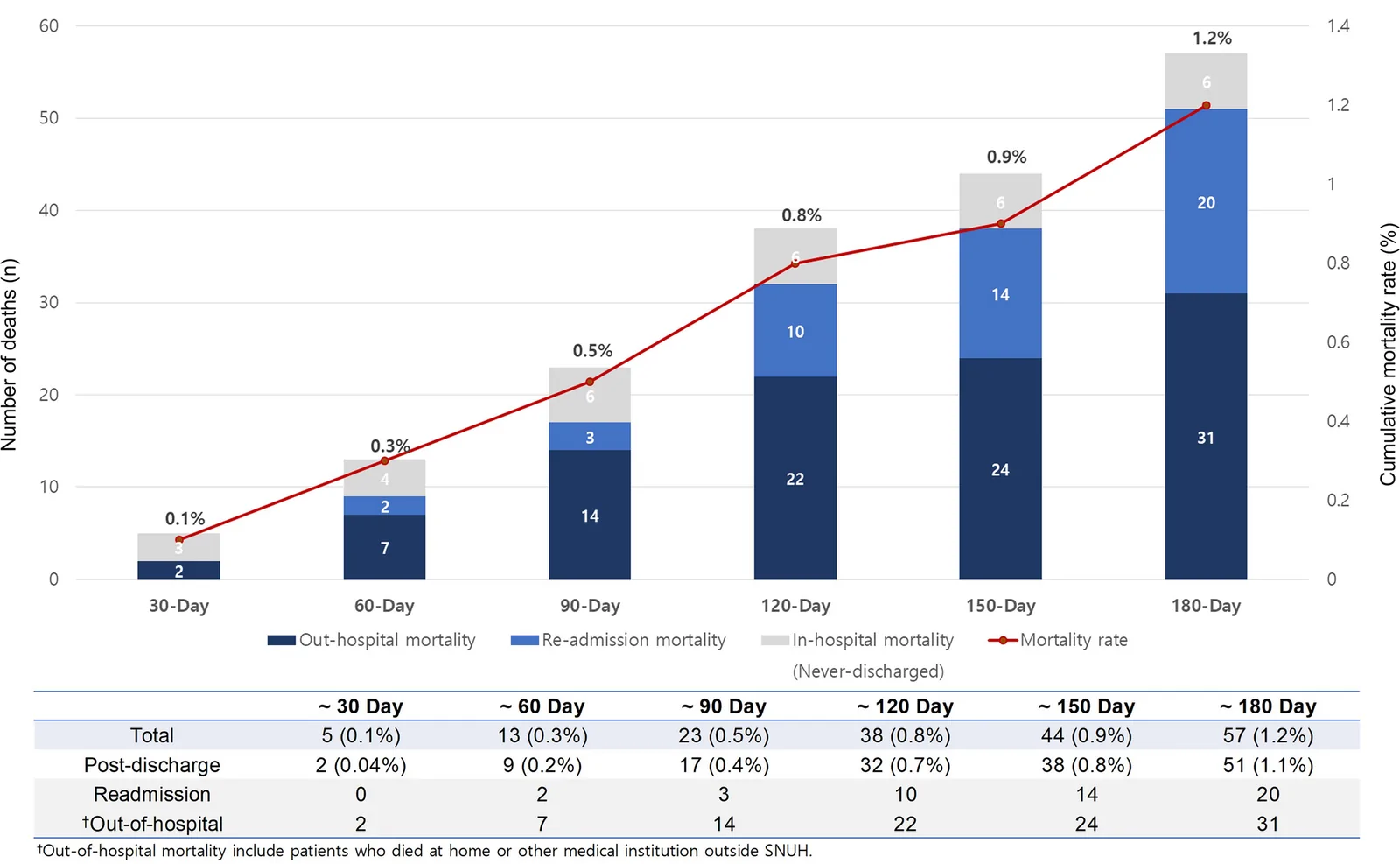
Cumulative 30-day interval postoperative mortality up to 6 months after gastrectomy

**Fig. 2:**
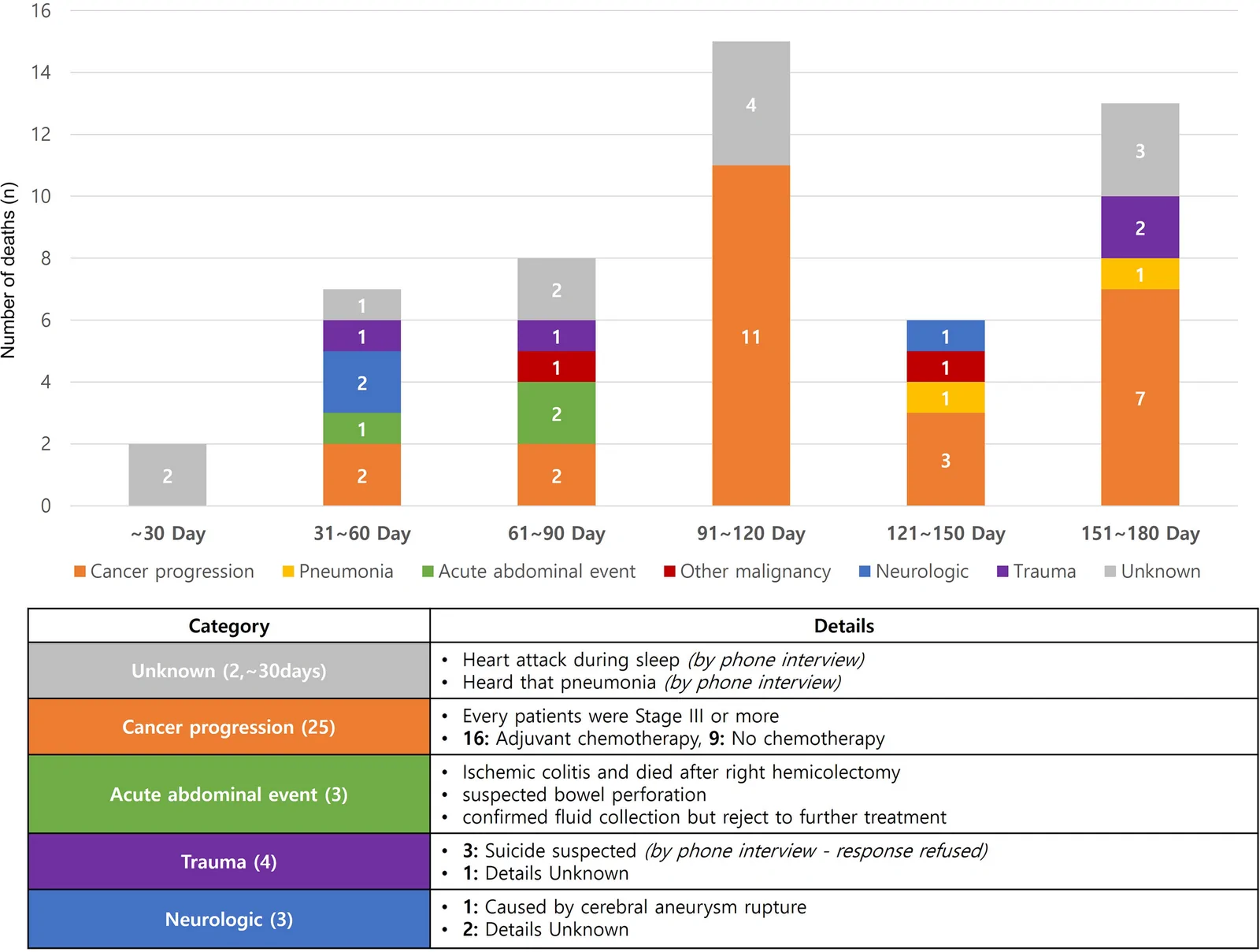
Causes of post-discharge mortality at 30-day intervals within 6 months after gastrectomy

**Fig. 3:**
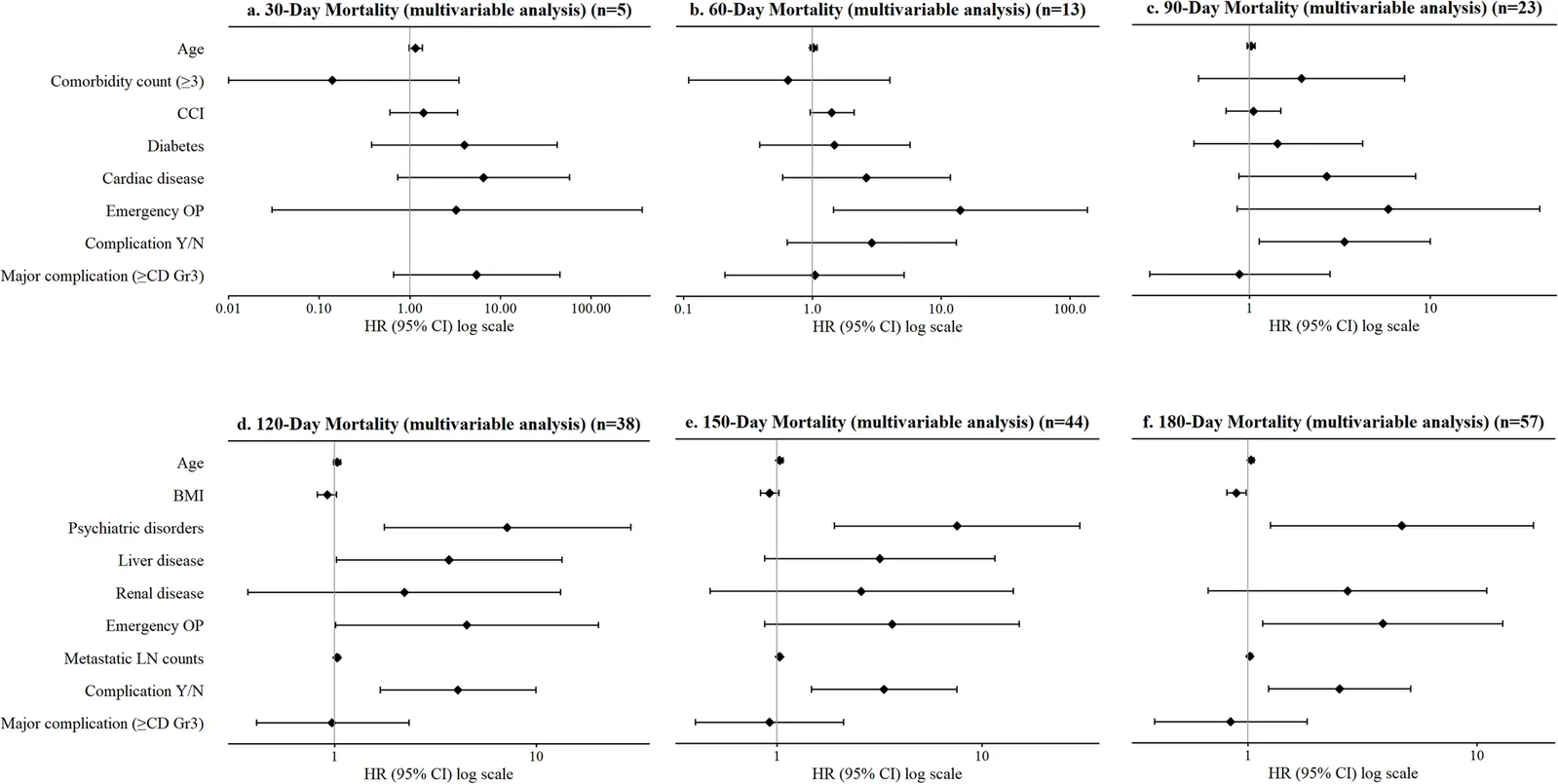
Time-specific risk factors for postoperative mortality within 6 months after gastrectomy

**Fig. 4:**
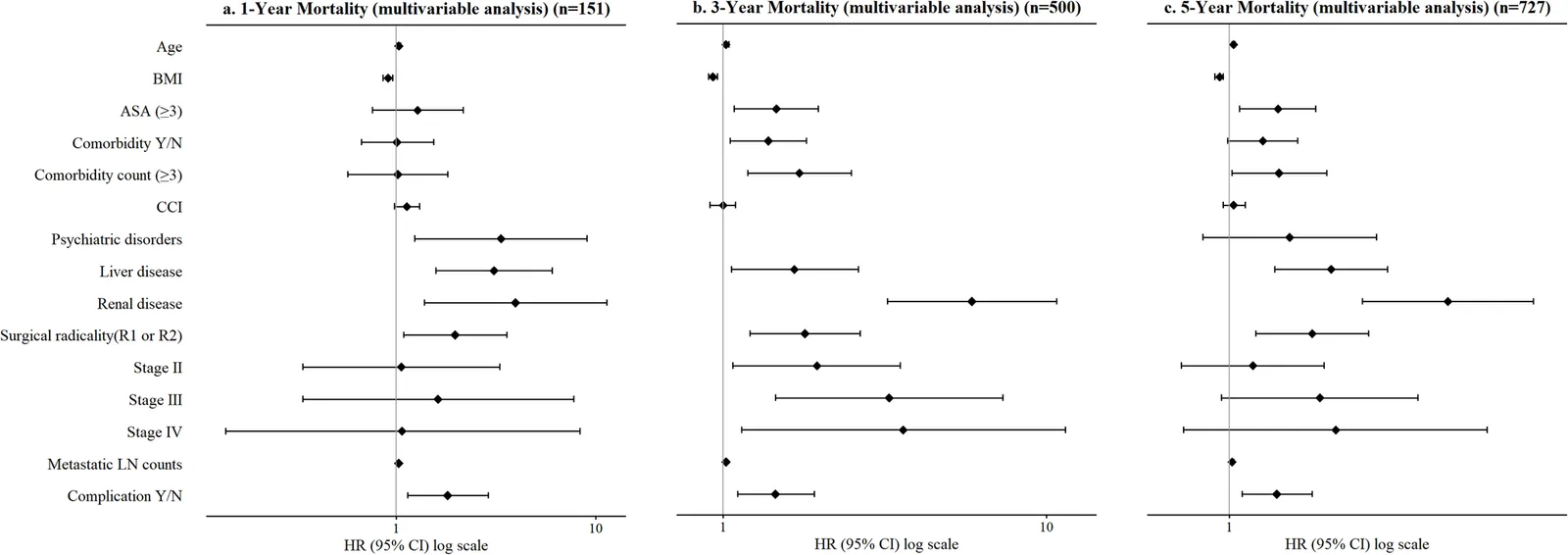
Long-term risk factors for postoperative mortality at 1-, 3-, and 5-year timepoints

**Fig. 5:**
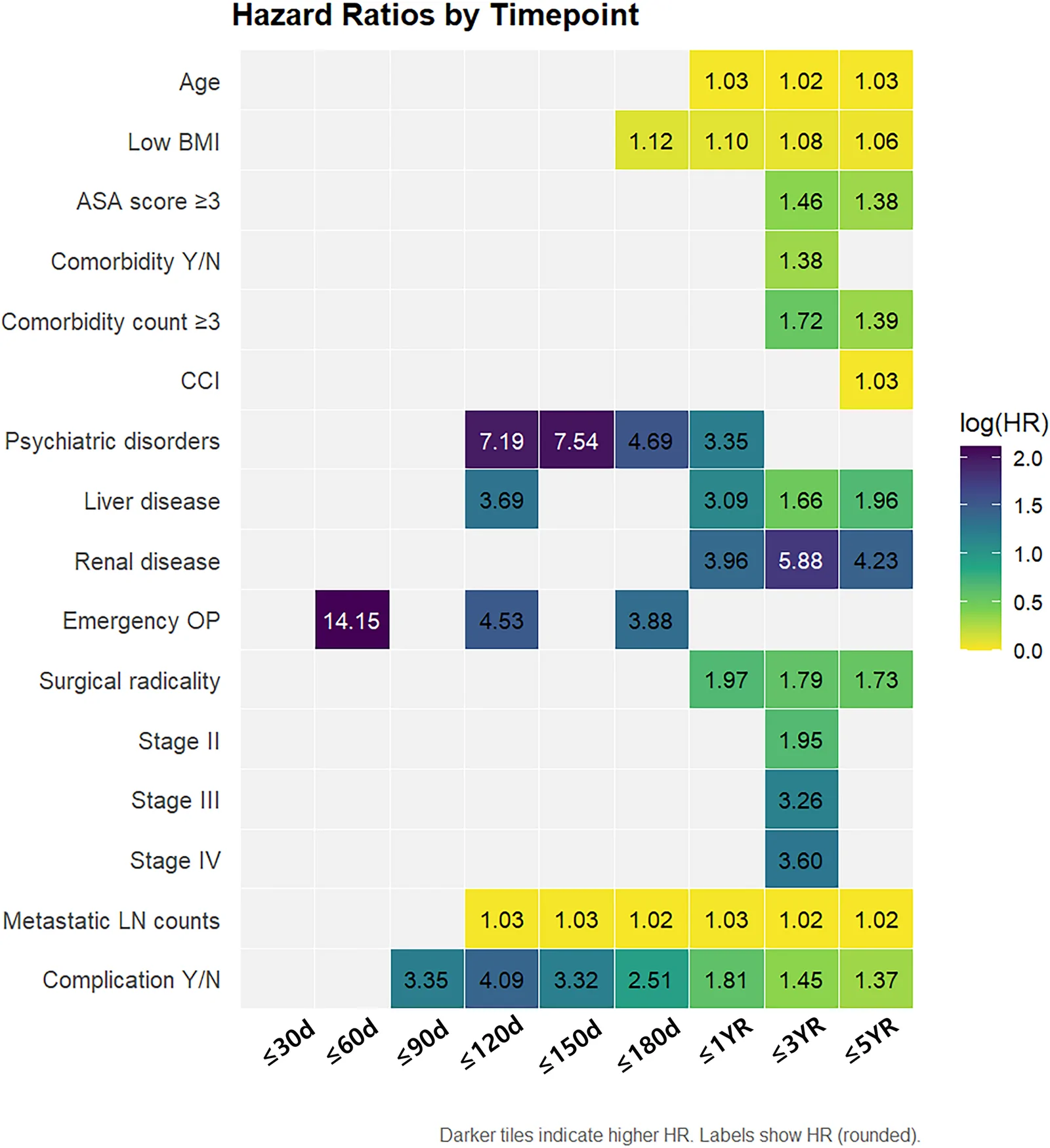
Heatmap summarizing time-specific hazard ratios up to 5 years after surgery

**Table 1:** Clinicopathologic characteristics of patients

Variable	Total (*N* = 4802)
***Patient’s characteristics***	
Age	62.0 (IQR: 54–70)
Sex	
Male	3036 (63.2%)
Female	1766 (36.8%)
BMI	24.0 (SD = 3.2)
ASA	
1	1795 (37.4%)
2	2767 (57.6%)
≥ 3	240 (5.0%)
***Operation***	
Elective operation	4785 (99.6%)
Emergency operation	17 (0.4%)
Extent of gastrectomy	
TG	970 (20.2%)
DG	2818 (58.7%)
PG	141 (3.0%)
PPG	873 (18.2%)
Surgical approach	
Open	1251 (26.1%)
Laparoscopy	3173 (66.1%)
Robot	378 (7.9%)
Lymph node dissection	
D1 or D1+	2897 (60.3%)
D2 or more	1905 (39.7%)
Neoadjuvant chemotherapy	
Upfront surgery	4772 (98.3%)
Conversion surgery	80 (1.7%)
Combined resection	
No	4022 (83.8%)
Yes	780 (16.2%)
Surgical radicality	
R0	4717 (98.2%)
R1 or R2	85 (1.8%)
***Pathology***	
TNM stage	
No residual tumor (pCR)	12 (0.2%)
I	3311 (69.0%)
II	655 (13.6%)
III	709 (14.8%)
IV	115 (2.4%)

BMI body mass index; ASA American Society of Anesthesiologists physical status classification; TG total gastrectomy; DG distal gastrectomy; PG proximal gastrectomy; PPG pylorus-preserving gastrectomy; EGC early gastric cancer; AGC advanced gastric cancer; pCR pathologic complete response

**Table 2:** Comorbidity and complications of patients

Variable	Total (*N* = 4802)
***Comorbidity***	
Number of comorbid conditions	
0	2398 (49.9%)
1	1401 (29.2%)
2	691 (14.4%)
≥ 3	312 (6.5%)
CCI	4 (SD = 2)
Comorbid condition	
Hypertension	1487 (30.9%)
Diabetes	777 (16.2%)
Cardiac disease	379 (7.9%)
Vascular disease	44 (0.9%)
Pulmonary disease	196 (4.1%)
Neurologic disease	60 (1.2%)
Psychiatric disorders	53 (1.1%)
Liver disease	103 (2.1%)
Renal disease	31 (0.6%)
Previous malignancy	368 (7.7%)
***Complication***	
Clavien–Dindo	
I	198 (4.1%)
II	451 (9.4%)
III	487 (10.1%)
IV	76 (1.6%)
V (30-Day + In-hospital mortality)	8 (0.17%)
**Surgical complications**	
Wound	135 (2.8%)
Fluid collection	273 (5.7%)
Intraabdominal bleeding	40 (0.8%)
Luminal bleeding	29 (0.6%)
Stenosis	137 (2.9%)
Motility disorder	117 (2.4%)
GI leak	127 (2.6%)
Other fistula	17 (0.4%)
Ischemia	9 (0.2%)
Reflux	2 (0.0%)
**Medical complications**	
Pulmonary	222 (4.6%)
Urinary	40 (0.8%)
Renal	8 (0.2%)
Hepatobiliary	30 (0.6%)
GI	29 (0.6%)
Other infection	44 (0.9%)
Cardiac	24 (0.5%)
Neuropsychic	33 (0.7%)
Vascular	26 (0.5%)
Endocrine	1 (0.0%)
Undetermined	53 (1.1%)

CCI Charlson Comorbidity Index; GI gastrointestinal

### Ethical approval

This study was approved by the Institutional Review Board of Seoul National University Hospital (IRB No. 2504-024-1627; July 15, 2025). The need for informed consent was waived due to the retrospective design using anonymized data, and all procedures complied with the Declaration of Helsinki.

## **Results**

### Baseline characteristics

Between January 2013 and September 2019, 4,802 patients underwent gastrectomy for primary gastric cancer at Seoul National University Hospital (Table 1). The median age was 62 years (IQR 54–70), and 63.2% were male. Most patients had ASA 1–2 (95%), and mean BMI was 24.0 ± 3.2.

Distal gastrectomy was most common (58.7%), followed by total (20.2%), pylorus-preserving (18.2%), and proximal (3.0%) gastrectomy. Minimally invasive approaches (laparoscopic or robotic) accounted for > 70%. Emergency operations were rare, accounting for 17 cases (0.4%).

Pathologically, 65.1% had early and 34.9% advanced gastric cancer; nodal metastasis was present in 29.9%. Stage distribution (AJCC 8th): I 69.0%, II 13.6%, III 14.8%, IV 2.4%.

Conversion surgery accounted for 1.7% of cases,* reflecting its selective use in patients with initially unresectable disease after response to systemic therapy.*

### Comorbidities and complications

Overall, 53.5% of patients had one or more comorbidities, and 6.5% had three or more (Table 2). The mean Charlson Comorbidity Index was 4.0. Hypertension (30.9%) and diabetes (16.2%) were most frequent, followed by cardiac disease (7.9%) and prior malignancy (7.7%), while psychiatric disorders were present in 53 patients (1.1%), predominantly mood disorders (*n* = 32), mainly depressive disorders (*n* = 29) with bipolar disorder in 3 patients, followed by anxiety-related disorders (*n* = 11), whereas psychotic disorders (*n* = 4), sleep disorders requiring medication (*n* = 4), and alcohol use disorder (*n* = 2) were less frequent.

Postoperative complications occurred in 25.2% of patients, of which 14.0% were major (Clavien–Dindo grade ≥ III). Eight patients (0.17%) died within 30 days or during hospitalization (Clavien–Dindo grade V). The most common surgical complications were fluid collection (5.7%), stenosis (2.9%), wound problems (2.8%), and leakage (2.6%), while pulmonary complications were the most frequent medical events (4.6%).

### Postoperative mortality rates

Within 6 months after gastrectomy, 57 patients (1.2%) died. Thirty-day mortality was 0.1% (*n* = 5), whereas 90-day mortality reached 0.5% (*n* = 23). Figure 1 shows cumulative deaths at 30-day intervals, classified as post-discharge, readmission, and out-of-hospital deaths, including those occurring at home or other institutions.

Of the five deaths within 30 days, two occurred after discharge and were identified only through National Statistical Office linkage. Including all in-hospital deaths among patients who never left the hospital, *the 30-day or in-hospital mortality (Clavien–Dindo grade V)*,* based on the traditional reporting definition used in routine clinical practice*, was 0.17% (*n* = 8). This was slightly higher than the crude 30-day mortality of 0.1% and reflects the inclusion of deaths that would otherwise have been missed after discharge.

Traditionally, deaths after readmission beyond 30 days were excluded from postoperative mortality. However, by including these cases, the 90-day mortality was nearly fivefold higher than the 30-day rate, confirming that short-term mortality endpoints based solely on early postoperative periods markedly underestimate true risk. Among post-discharge deaths, 2 occurred within 30 days, 6 within 60 days, and 17 within 90 days. By 6 months, 51 of 57 deaths (89.5%) had occurred after discharge, showing a steady accumulation of late postoperative mortality (Fig. 1).

### Causes of death

Eight patients met the definition of Clavien–Dindo grade V (30-day or in-hospital death). Two died after discharge, with unclear causes classified as unknown, though cardiopulmonary events were suspected based on family interviews.

Among the remaining six patients who died during hospitalization, most were elderly and had multiple comorbidities. Three deaths were attributed to surgical causes, including postoperative gastrointestinal leakage and procedure-related complications, while three were due to medical causes such as cardiopulmonary events or septic shock. These findings indicate that early postoperative mortality was not solely driven by technical surgical failure but was also strongly associated with underlying patient vulnerability and medical complications. Most deaths occurred within 90 days. Detailed information is provided in Supplementary Table 1.

Among 51 post-discharge deaths within six months, the pattern of causes shifted over time (Fig. 2). Two early cases remained unknown but were presumed cardiopulmonary, suggesting that medical causes may have contributed to a proportion of early post-discharge mortality. Beyond 90 days, cancer progression became the predominant cause, consistent with the natural course of advanced disease.

Three cases were attributed to acute abdominal events suggesting delayed surgical complications.

- A 70-year-old man without comorbidities underwent laparoscopic distal gastrectomy for pT1bN0 disease. After postoperative ileus and wound complications, he was discharged on POD 20 but readmitted 10 days later with abdominal pain. He underwent extended right hemicolectomy for ischemic colitis and died on POD 54 from unexplained cardiopulmonary arrest.
- A 73-year-old man with cardiac, pulmonary, and diabetic comorbidities received neoadjuvant chemotherapy for suspected pancreatic invasion, followed by conversion open total gastrectomy (ypT1bN0). After postoperative pulmonary complications and intra-abdominal fluid collection, he was discharged on POD 36 but readmitted with altered mental status. Gastrointestinal bleeding and suspected small bowel perforation were identified, and he died on POD 68 without further surgery.
- A 67-year-old woman without comorbidities underwent palliative open distal gastrectomy (R1 resection) for peritoneal seeding (pT4aN2M1). Postoperative intra-abdominal fluid collection was managed with percutaneous catheter drainage (PCD). She re-presented after discharge with recurrent fluid collection and wound discharge, declined further treatment and died on POD 71.

These cases underscore the possibility of late-onset surgical complications contributing to delayed mortality.

Four trauma-related deaths lacked definitive documentation; interviews suggested possible suicide in three cases. Three neurologic deaths were recorded—one from aneurysmal rupture and two from intracranial hemorrhage—though detailed clinical data were unavailable.

Collectively, these findings highlight the temporal evolution of postoperative mortality causes: early deaths dominated by surgical or cardiopulmonary complications and later deaths increasingly due to oncologic progression and systemic deterioration, reflecting a shift from perioperative factors to disease-related and systemic causes over time.

### Time-specific risk factors

Univariable and multivariable Cox proportional hazards regression analyses were performed in 30-day intervals up to 6 months, and at 1-, 3-, and 5-year timepoints. Full results are presented in Supplementary Tables 2–5. Variables that remained significant in multivariable analyses are summarized in a log-scale forest plot (Figs. 3 and 4). To facilitate visual comparison across time windows, all adjusted estimates (including the long-term results) are synthesized in a hazard-ratio heatmap (Fig. 5).

Up to 90 days after surgery, the presence of any postoperative complication—regardless of Clavien–Dindo grade—was the dominant determinant of mortality, conferring more than a threefold increased risk. This adverse effect persisted through 6 months, with more than a twofold increase in mortality risk, and remained detectable even at 5 years, although attenuated.

By approximately 120 days, comorbidity-related factors began to emerge as major contributors to mortality. Notably, psychiatric disorders were associated with a strikingly elevated risk, exceeding sevenfold at 120 days and remaining significant through 1 year. At the same time, hepatic disease also became an independent predictor, and the influence of metastatic lymph-node burden showed a consistent and sustained association from this stage onward, reflecting the growing oncologic contribution to late mortality.

By 180 days, nutritional and constitutional factors became increasingly relevant. Low BMI newly emerged as an independent risk factor, with each 1 kg/m² increase in BMI corresponding to an approximately 11% reduction in mortality risk. From the 1-year time point, chronological age itself became a significant determinant of survival, indicating a shift toward systemic and demographic vulnerability beyond the early postoperative phase.

At longer follow-up, oncologic burden and cumulative physiologic reserve dominated outcomes. At 3 years, pathologic TNM stage demonstrated a clear linear association with mortality, while at 5 years, advanced age, low BMI, high ASA score, multiple comorbidities, elevated CCI, liver and renal disease, non-R0 resection, metastatic lymph-node burden, and postoperative complications continued to exert persistent—although more modest—effects on long-term survival.

Collectively, these analyses illustrate a progressive transition in the drivers of postoperative mortality, from perioperative and complication-related factors in the early phase, to psychosocial, nutritional, systemic, and ultimately oncologic determinants as follow-up lengthens.

## Discussion

This study demonstrates that conventional 30-day mortality substantially underestimates the true burden of postoperative death after gastrectomy for gastric cancer. In our cohort, the crude 30-day mortality was 0.1%, and 30-day or in-hospital mortality was 0.17%, yet nearly 90% of deaths within the first six months occurred after hospital discharge, indicating that a large proportion of clinically meaningful mortality remains invisible when assessment is restricted to in-hospital or 30-day outcomes [19, 20, 25].

To move toward minimizing preventable postoperative mortality after gastric cancer surgery, it is therefore essential to identify patients who remain at risk of dying beyond the early postoperative window—particularly those who die after discharge. By extending follow-up and integrating national death registry data, our study sought to characterize these overlooked deaths and to determine which patients remain vulnerable after leaving the hospital. Our findings suggest that immediate postoperative mortality is largely related to perioperative stressors such as emergency surgery and complications [26, 27], whereas delayed mortality increasingly reflects patient frailty, systemic comorbidity, and oncologic progression.

Importantly, postoperative complications—regardless of severity—were associated with markedly increased mortality risk, persisting well beyond the index hospitalization and remaining detectable even at long-term follow-up [28–31]. These findings indicate that discharge does not signify recovery, but rather the beginning of a prolonged period of physiologic fragility. Patients who experience complicated postoperative courses therefore represent a critical target population for intensified post-discharge surveillance and intervention.

Beyond the early recovery phase, deaths occurring after discharge were associated with psychosocial, nutritional, systemic, and oncologic factors. Psychiatric comorbidity showed a strong association with mid-term mortality, suggesting that limited adherence, reduced resilience, and impaired follow-up engagement may partly explain this relationship, although a causal link cannot be established [32–34]. Nutritional vulnerability, reflected by low body mass index [35–37], and advancing age further shaped long-term outcomes [24, 38], consistent with accumulating systemic frailty. Ultimately, oncologic burden—including lymph-node metastasis and incomplete resection—remained strongly associated with survival, underscoring that long-term prognosis is governed by both tumor biology and host vulnerability.

In addition, systemic comorbidities—particularly liver and renal disease—were associated with delayed mortality, potentially through impaired metabolic homeostasis and reduced tolerance to adjuvant therapy [39, 40]. The cumulative burden of disease, as reflected by higher ASA scores, multiple comorbidities, and elevated Charlson Comorbidity Index, further amplified long-term risk. At the same time, surgical radicality remained a critical determinant of survival: patients with incomplete (non-R0) resection experienced persistently worse outcomes, underscoring the importance of complete oncologic clearance whenever feasible [41–43]. Ultimately, these findings highlight that long-term prognosis after gastrectomy is governed not solely by tumor biology, but by the integrated effects of oncologic, physiologic, and systemic vulnerability.

From a clinical perspective, our findings indicate that patients at high risk of delayed postoperative mortality require a fundamentally different survivorship strategy after surgery. Rather than relying on routine postoperative schedules, these patients may benefit from more frequent and structured follow-up, earlier detection of clinical deterioration, and timely referral to specialized care teams. Patients undergoing emergency surgery or experiencing postoperative complications warrant particularly close surveillance for at least six months, when the majority of early post-discharge deaths occur. In addition, patients with psychiatric comorbidity may benefit from coordinated multidisciplinary care beginning in the preoperative period and extending through the first postoperative year to support adherence and psychosocial resilience. Sustained nutritional intervention should also be prioritized, especially for patients with low body mass index or malnutrition, given the growing importance of nutritional vulnerability in late mortality. Finally, maximal oncologic radicality should be pursued whenever feasible, in light of its persistent prognostic significance throughout long-term follow-up. These findings also highlight the importance of preoperative risk stratification and optimization, including prehabilitation and multidisciplinary preparation, to reduce postoperative vulnerability [44, 45].

Collectively, these observations emphasize that postoperative mortality after gastrectomy is not a single perioperative event, but a dynamic process extending well beyond hospital discharge. Patients who die after 30 days—or after leaving the hospital—are not random; rather, they represent a clinically identifiable population defined by surgical stress, postoperative complications, psychosocial vulnerability, nutritional depletion, systemic comorbidity, and oncologic burden. Recognition of these risk profiles provides a concrete opportunity for prevention. Enhanced post-discharge surveillance, early rehabilitation, proactive psychiatric support, sustained nutritional intervention, and strict oncologic control may together *help reduce preventable mortality* after gastric cancer surgery.

Several limitations should be acknowledged. This was a retrospective, single-center analysis, and the number of early postoperative deaths was relatively small. Only overall survival was analyzed, as our primary objective was to capture the total burden of postoperative mortality rather than cancer-specific outcomes alone. Unmeasured confounders—including frailty status, socioeconomic conditions, and adherence to adjuvant therapy—could not be fully assessed.

 Although our observed 30-day mortality rate was lower than the nationwide average reported in Korea [14], previous large-scale studies from high-volume gastric cancer centers have similarly demonstrated very low perioperative mortality [46], suggesting that our results are broadly consistent with contemporary outcomes in specialized centers. This study was conducted at a high-volume tertiary referral center with a substantial proportion of patients with advanced disease and significant comorbidities, which may influence both patient selection and postoperative outcomes. No major institutional perioperative protocol changes occurred during the study period, reducing the likelihood that temporal practice variation confounded the observed mortality patterns. Nevertheless, caution is warranted when generalizing these findings to lower-volume institutions or settings with higher baseline mortality, and external validation in diverse clinical environments is needed.

In conclusion, postoperative mortality after gastrectomy extends far beyond the conventional 30-day window and is driven by identifiable risk factors that evolve over time. Focusing exclusively on early endpoints risks overlooking patients who remain vulnerable after discharge. By recognizing and addressing the determinants of post-discharge death, surgeons and care teams can move closer to achieving meaningful reductions in postoperative mortality after gastric cancer surgery.

## Electronic Supplementary Material

Below is the link to the electronic supplementary material.

Supplementary Material
