# Supplementary material for: Beyond 30-day mortality: causes and risk factors of half-year postdischarge mortality after gastric cancer surgery
Source: Gastric Cancer. 2026 Oct 21;29(6):1360–71. doi: 10.1007/s10120-026-01750-x (PMC13754945; doi:10.1007/s10120-026-01750-x)
Supplement: Supplementary file 1 [file 10120_2026_1750_MOESM1_ESM.docx]

**Supplementary Tables**

**Supplementary Table 1.** Clinical characteristics and causes of 30-day and in-hospital mortality cases (Clavien–Dindo grade V)

| **Time of death** | **Age/Sex** | **Comorbidity** | **CCI** | **Approach** | **OP** | **Stage** | **cause of death** | **Details** | **Category** | |
| --- | --- | --- | --- | --- | --- | --- | --- | --- | --- | --- |
| POD#6 | 71/M | 2VD  s/p PCI | 5 | Lap. | PPG | IB | postOP AMI | postOP AMI | | Medical |
| POD#19 | 84/M | DM, HTN s/p CABG h/o Stroke | 9 | Open | TG | IIIC | postOP Cardiac complication | Patient underwent CABG using RtGEA -> Rt.GEA ligation during OP | | Surgical |
| POD#21 | 73/M | HTN | 5 | Lap. | DG | IA | Unknown | Unknown | | Unknown |
| POD#24 | 83/M | DM | 8 | Lap. | DG | IIA | Unknown | Unknown | | Unknown |
| POD#27 | 91/M | DM, HTN, CKD,  Angina | 11 | Open | DG | IV | postOP Pulmonary complication | Respiratory arrest  after chest PCD | | Medical |
| POD#51 | 39/M | Paroxysmal Nocturnal Hemoglobinuria | 4 | Lap. | TG | IA | Refractory septic shock | Refractory septic shock  d/t Bone marrow dysfunction | | Medical |
| POD#65 | 70/M | Alcoholic LC,  Esophageal cancer | 4 | Open | TG | IIIA | postOP GI leak | Synchronous esophageal cancer s/p colon interposition  colo-colostomy leakage & interpositioned colon - bronchus fistula | | Surgical |
| POD#75 | 59/M | DM, HTN ESRD s/p K-TPL | 6 | Open | TG | IIIC | postOP GI leak | Jejunal stump leak  -> liver failure, refractory septic shock | | Surgical |

POD postoperative day; 2VD two-vessel disease; PCI percutaneous coronary intervention; DM diabetes mellitus; HTN hypertension; CABG coronary artery bypass grafting; h/o history of; RtGEA right gastroepiploic artery; CKD chronic kidney disease; ESRD end-stage renal disease; K-TPL kidney transplantation; AMI acute myocardial infarction; PCD percutaneous catheter drainage; LC liver cirrhosis; DG distal gastrectomy; TG total gastrectomy; PPG pylorus-preserving gastrectomy; Lap. laparoscopic; OP operation; CCI Charlson Comorbidity Index; GI gastrointestinal.

**Supplementary Table 2.** Univariable and multivariable Cox proportional hazards regression analyses of risk factors for 30-day mortality

| **30-Day Mortality** (n= 5) | Univariable | | | |  | Multivariable | | | |
| --- | --- | --- | --- | --- | --- | --- | --- | --- | --- |
|  | HR | 95% CI of HR | | *p*-value |  | HR | 95% CI of HR | | *p*-value |
|  |  |  |  |  |  |  |  |  |  |
| ***Patient’s characteristics*** |  |  |  |  |  |  |  |  |  |
| Age | 1.25 | 1.11 | 1.41 | **<0.001** |  | 1.16 | 0.98 | 1.38 | 0.081 |
| Sex (Male) | 42.13 | 0.02 | 8.E+04 | 0.332 |  |  |  |  |  |
| BMI | 1.01 | 0.77 | 1.32 | 0.935 |  |  |  |  |  |
| ASA (≥3) | 4.76 | 0.53 | 42.61 | 0.163 |  |  |  |  |  |
| ***Comorbidity*** |  |  |  |  |  |  |  |  |  |
| Comorbidity Y/N | 4 | 0.45 | 35.75 | 0.215 |  |  |  |  |  |
| Comorbidity count (≥3) | 9.61 | 1.61 | 57.51 | **0.013** |  | 0.14 | 0.01 | 3.47 | 0.232 |
| CCI | 1.9 | 1.47 | 2.45 | **<0.001** |  | 1.42 | 0.6 | 3.37 | 0.432 |
| Hypertension | 3.35 | 0.56 | 20.02 | 0.186 |  |  |  |  |  |
| Diabetes | 7.78 | 1.3 | 45.54 | **0.025** |  | 4.01 | 0.38 | 42.15 | 0.247 |
| Cardiac disease | 17.57 | 2.94 | 105.14 | **0.002** |  | 6.51 | 0.73 | 57.98 | 0.093 |
| Pulmonary disease | 0.047 | 0 | 2.E+07 | 0.76 |  |  |  |  |  |
| Vascular disease | 0.05 | 0 | 3.63E+16 | 0.886 |  |  |  |  |  |
| Neurologic disease | 0.049 | 0 | 1.01E+14 | 0.867 |  |  |  |  |  |
| Psychiatric disorders | 22.65 | 2.53 | 202.66 | **0.005** |  | 18.75 | 0.87 | 405.31 | 0.062 |
| Liver disease | 0.048 | 0 | 2.5466E+10 | 0.826 |  |  |  |  |  |
| Renal disease | 0.049 | 0 | 9.19E+19 | 0.904 |  |  |  |  |  |
| ***Operation*** |  |  |  |  |  |  |  |  |  |
| Emergency OP | 70.34 | 7.86 | 629.31 | **<0.001** |  | 3.24 | 0.03 | 366.5 | 0.626 |
| Lymph node dissection (D2) | 6.09 | 0.68 | 54.44 | 0.106 |  |  |  |  |  |
| Surgical radicality (R1 or R2) | 14.04 | 1.57 | 125.58 | **0.018** |  | 3.75 | 0 | 8.E+03 | 0.735 |
| ***Pathology*** |  |  |  |  |  |  |  |  |  |
| EGC/AGC | 7.45 | 0.83 | 66.68 | 0.072 |  |  |  |  |  |
| **T-stage** |  |  |  |  |  |  |  |  |  |
| T1 | Ref |  |  |  |  | Ref |  |  |  |
| T2 | 11.82 | 1.07 | 130.39 | **0.044** |  | 6.92 | 0.42 | 115.54 | 0.178 |
| T3 | 5.22 | 0.33 | 83.5 | 0.242 |  | 0.82 | 0.01 | 138.53 | 0.938 |
| T4 | 5.63 | 0.35 | 90.06 | 0.222 |  | 0.09 | 0 | 217.15 | 0.547 |
| Lymph node metastasis | 3.52 | 0.59 | 21.05 | 0.168 |  |  |  |  |  |
| M stage | 11.07 | 1.24 | 99.05 | **0.032** |  | 4.27 | 0 | 5.3098E+10 | 0.903 |
| **TNM Stage** |  |  |  |  |  |  |  |  |  |
| Stage I | Ref |  |  |  |  | Ref |  |  |  |
| Stage II | 2.53 | 0.23 | 27.87 | 0.449 |  | 1.12 | 0.06 | 20.62 | 0.941 |
| Stage III | 2.34 | 0.21 | 25.76 | 0.488 |  | 2.52 | 0.02 | 281.59 | 0.701 |
| Stage IV | 14.39 | 1.31 | 158.7 | **0.029** |  | 0.57 | 0 | 8885231977 | 0.963 |
| Metastatic LN counts | 1.03 | 0.97 | 1.11 | 0.335 |  |  |  |  |  |
| ***Complication*** |  |  |  |  |  |  |  |  |  |
| Complication Y/N | 5.44 | 0.91 | 32.55 | 0.064 |  |  |  |  |  |
| Major complication (≥CD Gr3) | 9.21 | 1.54 | 55.09 | **0.015** |  | 5.47 | 0.66 | 45.01 | 0.114 |

HR, hazard ratio; CI, confidence interval; BMI, body mass index; ASA, American Society of Anesthesiologists physical status classification; CCI, Charlson Comorbidity Index; OP, operation; EGC, early gastric cancer; AGC, advanced gastric cancer; LN, lymph node; TNM, tumor–node–metastasis; CD Gr, Clavien–Dindo Grade.

**Supplementary Table 3.** Univariable and multivariable Cox proportional hazards regression analyses of risk factors for 90-day mortality

| **90-Day Mortality** (n= 23) | Univariable | | | |  | Multivariable | | | |
| --- | --- | --- | --- | --- | --- | --- | --- | --- | --- |
|  | HR | 95% CI of HR | | *p*-value |  | HR | 95% CI of HR | | *p*-value |
|  |  |  |  |  |  |  |  |  |  |
| ***Patient’s characteristics*** |  |  |  |  |  |  |  |  |  |
| Age | 1.06 | 1.02 | 1.12 | **0.003** |  | 1.02 | 0.97 | 1.07 | 0.389 |
| Sex (Male) | 2.77 | 0.94 | 8.14 | 0.064 |  |  |  |  |  |
| BMI | 0.88 | 0.77 | 1.01 | 0.068 |  |  |  |  |  |
| ASA (≥3) | 2.87 | 0.85 | 9.64 | 0.089 |  |  |  |  |  |
| ***Comorbidity*** |  |  |  |  |  |  |  |  |  |
| Comorbidity Y/N | 1.88 | 0.8 | 4.42 | 0.151 |  |  |  |  |  |
| Comorbidity count (≥3) | 6.35 | 2.61 | 15.44 | **<0.001** |  | 1.94 | 0.52 | 7.21 | 0.322 |
| CCI | 1.55 | 1.32 | 1.83 | **<0.001** |  | 1.05 | 0.74 | 1.49 | 0.801 |
| Hypertension | 0.98 | 0.4 | 2.37 | 0.957 |  |  |  |  |  |
| Diabetes | 2.77 | 1.18 | 6.54 | **0.02** |  | 1.43 | 0.49 | 4.22 | 0.514 |
| Cardiac disease | 5.15 | 2.12 | 12.51 | **<0.001** |  | 2.68 | 0.87 | 8.31 | 0.087 |
| Pulmonary disease | 2.24 | 0.53 | 9.55E+00 | 0.276 |  |  |  |  |  |
| Vascular disease | 0.05 | 0 | 1.03E+07 | 0.758 |  |  |  |  |  |
| Neurologic disease | 0.05 | 0 | 666871.64 | 0.719 |  |  |  |  |  |
| Psychiatric disorders | 4.14 | 0.56 | 30.68 | 0.165 |  |  |  |  |  |
| Liver disease | 4.36 | 1.02 | 18.59 | **0.047** |  | 3.5 | 0.74 | 16.58 | 0.114 |
| Renal disease | 7.04 | 0.95 | 5.22E+01 | 0.056 |  |  |  |  |  |
| ***Operation*** |  |  |  |  |  |  |  |  |  |
| Emergency OP | 29.07 | 6.81 | 123.98 | **<0.001** |  | 5.86 | 0.85 | 40.41 | 0.073 |
| Lymph node dissection (D2) | 1.4 | 0.62 | 3.16E+00 | 0.424 |  |  |  |  |  |
| Surgical radicality (R1 or R2) | 12.04 | 4.1 | 35.39 | **<0.001** |  | 2.14 | 0.32 | 14.2 | 0.429 |
| ***Pathology*** |  |  |  |  |  |  |  |  |  |
| EGC/AGC | 3.5 | 1.48 | 8.26 | **0.004** |  | 0.41 | 0 | 6.E+06 | 0.915 |
| **T-stage** |  |  |  |  |  |  |  |  |  |
| T1 | Ref |  |  |  |  | Ref |  |  |  |
| T2 | 2.22 | 0.59 | 8.36 | 0.239 |  | 5.55 | 0 | 9.E+07 | 0.839 |
| T3 | 2.61 | 0.79 | 8.68 | 0.117 |  | 4.29 | 0 | 7.E+07 | 0.864 |
| T4 | 5.66 | 2.13 | 15.08 | **<0.001** |  | 4.62 | 0 | 8.E+07 | 0.857 |
| Lymph node metastasis | 2.56 | 1.13 | 5.81 | **0.024** |  | 0.49 | 0.07 | 3.63 | 0.483 |
| M stage | 9.5 | 3.23 | 27.92 | **<0.001** |  | 0.19 | 0.01 | 3.02 | 0.237 |
| **TNM Stage** |  |  |  |  |  |  |  |  |  |
| Stage I | Ref |  |  |  |  | Ref |  |  |  |
| Stage II | 1.01 | 0.22 | 4.61 | 0.989 |  | 0.67 | 0.04 | 11.24 | 0.779 |
| Stage III | 2.81 | 1.02 | 7.73 | **0.046** |  | 2.71 | 0.05 | 141.82 | 0.622 |
| Stage IV | 14.64 | 5 | 42.83 | **<0.001** |  | 19.59 | 0.19 | 1974.55 | 0.206 |
| Metastatic LN counts | 1.04 | 1.01 | 1.07 | **0.021** |  | 1 | 0.94 | 1.06 | 0.911 |
| ***Complication*** |  |  |  |  |  |  |  |  |  |
| Complication Y/N | 4.73 | 2.08 | 10.79 | **<0.001** |  | 3.35 | 1.13 | 9.97 | **0.03** |
| Major complication (≥CD Gr3) | 3.29 | 1.39 | 7.75 | **0.007** |  | 0.88 | 0.28 | 2.78 | 0.825 |

HR, hazard ratio; CI, confidence interval; BMI, body mass index; ASA, American Society of Anesthesiologists physical status classification; CCI, Charlson Comorbidity Index; OP, operation; EGC, early gastric cancer; AGC, advanced gastric cancer; LN, lymph node; TNM, tumor–node–metastasis; CD Gr, Clavien–Dindo Grade.

**Supplementary Table 4.** Univariable and multivariable Cox proportional hazards regression analyses of risk factors for 180-day mortality

| **180-Day Mortality** (n= 57) | Univariable | | | |  | Multivariable | | | |
| --- | --- | --- | --- | --- | --- | --- | --- | --- | --- |
|  | HR | 95% CI of HR | | *p*-value |  | HR | 95% CI of HR | | *p*-value |
|  |  |  |  |  |  |  |  |  |  |
| ***Patient’s characteristics*** |  |  |  |  |  |  |  |  |  |
| Age | 1.07 | 1.05 | 1.1 | **<0.001** |  | 1.03 | 1 | 1.06 | 0.086 |
| Sex (Male) | 1.63 | 0.91 | 2.65 | 0.103 |  |  |  |  |  |
| BMI | 0.83 | 0.76 | 0.91 | **<0.001** |  | 0.89 | 0.81 | 0.98 | **0.013** |
| ASA (≥3) | 4.1 | 2.07 | 8.12 | **<0.001** |  | 1.18 | 0.56 | 2.51 | 0.664 |
| ***Comorbidity*** |  |  |  |  |  |  |  |  |  |
| Comorbidity Y/N | 2.36 | 1.34 | 4.16 | **0.003** |  | 1.07 | 0.53 | 2.17 | 0.857 |
| Comorbidity count (≥3) | 5.24 | 2.91 | 9.45 | **<0.001** |  | 1.75 | 0.75 | 4.12 | 0.197 |
| CCI | 1.62 | 1.47 | 1.79 | **<0.001** |  | 1.25 | 0.99 | 1.56 | 0.059 |
| Hypertension | 1.12 | 0.64 | 1.93 | 0.698 |  |  |  |  |  |
| Diabetes | 2.03 | 1.14 | 3.62 | **0.016** |  | 1 | 0.49 | 2.06 | 0.997 |
| Cardiac disease | 3.15 | 1.67 | 5.96 | **<0.001** |  | 1.32 | 0.58 | 2.97 | 0.508 |
| Pulmonary disease | 2.27 | 0.91 | 5.67 | 0.081 |  |  |  |  |  |
| Vascular disease | 3.96 | 0.97 | 16.21 | 0.056 |  |  |  |  |  |
| Neurologic disease | 1.41 | 0.2 | 10.2 | 0.732 |  |  |  |  |  |
| Psychiatric disorders | 5.12 | 1.6 | 16.36 | **0.006** |  | 4.69 | 1.25 | 17.57 | **0.022** |
| Liver disease | 2.56 | 0.8 | 8.2 | 0.112 |  |  |  |  |  |
| Renal disease | 8.85 | 2.77 | 28.32 | **<0.001** |  | 2.72 | 0.67 | 10.99 | 0.161 |
| ***Operation*** |  |  |  |  |  |  |  |  |  |
| Emergency OP | 24.3 | 8.79 | 67.14 | **<0.001** |  | 3.88 | 1.16 | 12.94 | **0.028** |
| Lymph node dissection (D2) | 2.83 | 1.64 | 4.87 | **<0.001** |  | 1.28 | 0.68 | 2.42 | 0.439 |
| Surgical radicality (R1 or R2) | 11.02 | 5.41 | 22.46 | **<0.001** |  | 1.35 | 0.46 | 3.95 | 0.583 |
| ***Pathology*** |  |  |  |  |  |  |  |  |  |
| EGC/AGC | 7.87 | 4.08 | 15.19 | **<0.001** |  | 0.18 | 0 | 3.E+04 | 0.779 |
| **T-stage** |  |  |  |  |  |  |  |  |  |
| T1 | Ref |  |  |  |  | Ref |  |  |  |
| T2 | 2.69 | 0.94 | 7.75 | 0.066 |  | 11.35 | 0 | 2.E+06 | 0.695 |
| T3 | 4.29 | 1.78 | 10.36 | **0.001** |  | 9.56 | 0 | 2.E+06 | 0.717 |
| T4 | 16.78 | 8.46 | 33.28 | **<0.001** |  | 23.79 | 0 | 5.E+06 | 0.61 |
| Lymph node metastasis | 5.57 | 3.16 | 9.82 | **<0.001** |  | 0.53 | 0.13 | 2.21 | 0.384 |
| M stage | 11.05 | 5.73 | 21.34 | **<0.001** |  | 0.28 | 0.03 | 2.61 | 0.261 |
| **TNM Stage** |  |  |  |  |  |  |  |  |  |
| Stage I | Ref |  |  |  |  | Ref |  |  |  |
| Stage II | 1.81 | 0.65 | 5.02 | 0.256 |  | 0.99 | 0.13 | 7.37 | 0.991 |
| Stage III | 8.79 | 4.59 | 16.84 | **<0.001** |  | 2.93 | 0.18 | 47.99 | 0.452 |
| Stage IV | 25.86 | 11.96 | 55.91 | **<0.001** |  | 5.26 | 0.16 | 169.36 | 0.349 |
| Metastatic LN counts | 1.06 | 1.04 | 1.07 | **<0.001** |  | 1.02 | 1 | 1.04 | **0.017** |
| ***Complication*** |  |  |  |  |  |  |  |  |  |
| Complication Y/N | 4.08 | 2.42 | 6.85 | **<0.001** |  | 2.51 | 1.23 | 5.13 | **0.012** |
| Major complication (≥CD Gr3) | 3.1 | 1.79 | 5.38 | **<0.001** |  | 0.84 | 0.39 | 1.81 | 0.661 |

HR, hazard ratio; CI, confidence interval; BMI, body mass index; ASA, American Society of Anesthesiologists physical status classification; CCI, Charlson Comorbidity Index; OP, operation; EGC, early gastric cancer; AGC, advanced gastric cancer; LN, lymph node; TNM, tumor–node–metastasis; CD Gr, Clavien–Dindo Grade.

**Supplementary Table 5.** Univariable and multivariable Cox proportional hazards regression analyses of risk factors for 5-year mortality

| **5-Year Mortality** (n= 727) | Univariable | | | |  | Multivariable | | | |
| --- | --- | --- | --- | --- | --- | --- | --- | --- | --- |
|  | HR | 95% CI of HR | | *p*-value |  | HR | 95% CI of HR | | *p*-value |
|  |  |  |  |  |  |  |  |  |  |
| ***Patient’s characteristics*** |  |  |  |  |  |  |  |  |  |
| Age | 1.04 | 1.03 | 1.04 | **<0.001** |  | 1.03 | 1.02 | 1.04 | **<0.001** |
| Sex (Male) | 1.14 | 0.98 | 1.33 | 0.094 |  |  |  |  |  |
| BMI | 0.9 | 0.88 | 0.92 | **<0.001** |  | 0.94 | 0.91 | 0.96 | **<0.001** |
| ASA (≥3) | 2.56 | 2.03 | 3.24 | **<0.001** |  | 1.38 | 1.07 | 1.77 | **0.014** |
| ***Comorbidity*** |  |  |  |  |  |  |  |  |  |
| Comorbidity Y/N | 1.63 | 1.4 | 1.89 | **<0.001** |  | 1.25 | 0.99 | 1.57 | 0.063 |
| Comorbidity count (≥3) | 2.87 | 2.34 | 3.52 | **<0.001** |  | 1.39 | 1.02 | 1.9 | **0.036** |
| CCI | 1.42 | 1.37 | 1.48 | **<0.001** |  | 1.03 | 0.96 | 1.11 | **0.422** |
| Hypertension | 1.32 | 1.14 | 1.54 | **<0.001** |  | 0.86 | 0.7 | 1.07 | 0.176 |
| Diabetes | 1.45 | 1.21 | 1.73 | **<0.001** |  | 1 | 0.79 | 1.26 | 0.997 |
| Cardiac disease | 1.87 | 1.51 | 2.31 | **<0.001** |  | 1.03 | 0.79 | 1.35 | 0.835 |
| Pulmonary disease | 1.34 | 0.97 | 1.86 | 0.078 |  |  |  |  |  |
| Vascular disease | 2.66 | 1.62 | 4.36 | **<0.001** |  | 1.38 | 0.8 | 2.36 | 0.246 |
| Neurologic disease | 2.79 | 1.83 | 4.27 | **<0.001** |  | 1.49 | 0.96 | 2.31 | 0.076 |
| Psychiatric disorders | 1.89 | 1.12 | 3.21 | **0.018** |  | 1.49 | 0.84 | 2.64 | 0.173 |
| Liver disease | 2.94 | 2.12 | 4.07 | **<0.001** |  | 1.96 | 1.35 | 2.84 | **<0.001** |
| Renal disease | 4.83 | 2.94 | 7.93 | **<0.001** |  | 4.23 | 2.41 | 7.43 | **<0.001** |
| ***Operation*** |  |  |  |  |  |  |  |  |  |
| Emergency OP | 7.38 | 4.07 | 13.39 | **<0.001** |  | 1.05 | 0.56 | 1.98 | 0.887 |
| Lymph node dissection (D2) | 2.72 | 2.34 | 3.16 | **<0.001** |  | 1.17 | 0.98 | 1.39 | 0.079 |
| Surgical radicality (R1 or R2) | 14.21 | 11.09 | 18.21 | **<0.001** |  | 1.73 | 1.19 | 2.51 | **0.004** |
| ***Pathology*** |  |  |  |  |  |  |  |  |  |
| EGC/AGC | 6.42 | 5.44 | 7.59 | **<0.001** |  | 2.55 | 0.58 | 11.19 | 0.216 |
| **T-stage** |  |  |  |  |  |  |  |  |  |
| T1 | Ref |  |  |  |  | Ref |  |  |  |
| T2 | 2.24 | 1.7 | 2.95 | **<0.001** |  | 0.61 | 0.14 | 2.64 | 0.512 |
| T3 | 4.76 | 3.84 | 5.9 | **<0.001** |  | 0.76 | 0.18 | 3.19 | 0.708 |
| T4 | 14.63 | 12.2 | 17.55 | **<0.001** |  | 1.62 | 0.39 | 6.64 | 0.507 |
| Lymph node metastasis | 5.86 | 5.02 | 6.84 | **<0.001** |  | 1.24 | 0.89 | 1.72 | 0.205 |
| M stage | 15.66 | 12.53 | 19.57 | **<0.001** |  | 1.47 | 0.65 | 3.28 | 0.354 |
| **TNM Stage** |  |  |  |  |  |  |  |  |  |
| Stage I | Ref |  |  |  |  | Ref |  |  |  |
| Stage II | 2.79 | 2.2 | 3.52 | **<0.001** |  | 1.17 | 0.73 | 1.87 | 0.52 |
| Stage III | 9.36 | 7.85 | 11.17 | **<0.001** |  | 1.82 | 0.95 | 3.47 | 0.071 |
| Stage IV | 34.6 | 27.1 | 44.18 | **<0.001** |  | 2.02 | 0.74 | 5.48 | 0.169 |
| Metastatic LN counts | 1.06 | 1.06 | 1.07 | **<0.001** |  | 1.02 | 1.02 | 1.03 | **<0.001** |
| ***Complication*** |  |  |  |  |  |  |  |  |  |
| Complication Y/N | 2.05 | 1.76 | 2.39 | **<0.001** |  | 1.37 | 1.09 | 1.73 | **0.007** |
| Major complication (≥CD Gr3) | 1.88 | 1.58 | 2.25 | **<0.001** |  | 1.11 | 0.85 | 1.44 | 0.442 |

HR, hazard ratio; CI, confidence interval; BMI, body mass index; ASA, American Society of Anesthesiologists physical status classification; CCI, Charlson Comorbidity Index; OP, operation; EGC, early gastric cancer; AGC, advanced gastric cancer; LN, lymph node; TNM, tumor–node–metastasis; CD Gr, Clavien–Dindo Grade.
